# Prokaryotic evolution and the tree of life are two different things

**DOI:** 10.1186/1745-6150-4-34

**Published:** 2009-09-29

**Authors:** Eric Bapteste, Maureen A O'Malley, Robert G Beiko, Marc Ereshefsky, J Peter Gogarten, Laura Franklin-Hall, François-Joseph Lapointe, John Dupré, Tal Dagan, Yan Boucher, William Martin

**Affiliations:** 1 UPMC, UMR CNRS 7138, 75005 Paris, France; 2 ESRC Centre for Genomics in Society (Egenis), University of Exeter, Exeter, UK; 3 Faculty of Computer Science, Dalhousie University, Halifax, Nova Scotia, Canada; 4 Department of Philosophy, University of Calgary, Calgary, Alberta, Canada; 5 Department of Molecular and Cell Biology, University of Connecticut, USA; 6 Department of Philosophy, NYU, USA; 7 Département de sciences biologiques, Université de Montréal, Montréal (Québec), Canada; 8 Institute of Botany, University of Düsseldorf, Düsseldorf, Germany; 9 Department of Civil and Environmental Engineering, MIT, Cambridge, MA, USA

## Abstract

**Background:**

The concept of a tree of life is prevalent in the evolutionary literature. It stems from attempting to obtain a grand unified natural system that reflects a recurrent process of species and lineage splittings for all forms of life. Traditionally, the discipline of systematics operates in a similar hierarchy of bifurcating (sometimes multifurcating) categories. The assumption of a universal tree of life hinges upon the process of evolution being tree-like throughout all forms of life and all of biological time. In multicellular eukaryotes, the molecular mechanisms and species-level population genetics of variation do indeed mainly cause a tree-like structure over time. In prokaryotes, they do not. Prokaryotic evolution and the tree of life are two different things, and we need to treat them as such, rather than extrapolating from macroscopic life to prokaryotes. In the following we will consider this circumstance from philosophical, scientific, and epistemological perspectives, surmising that phylogeny opted for a single model as a holdover from the Modern Synthesis of evolution.

**Results:**

It was far easier to envision and defend the concept of a universal tree of life before we had data from genomes. But the belief that prokaryotes are related by such a tree has now become stronger than the data to support it. The monistic concept of a single universal tree of life appears, in the face of genome data, increasingly obsolete. This traditional model to describe evolution is no longer the most scientifically productive position to hold, because of the plurality of evolutionary patterns and mechanisms involved. Forcing a single bifurcating scheme onto prokaryotic evolution disregards the non-tree-like nature of natural variation among prokaryotes and accounts for only a minority of observations from genomes.

**Conclusion:**

Prokaryotic evolution and the tree of life are two different things. Hence we will briefly set out alternative models to the tree of life to study their evolution. Ultimately, the plurality of evolutionary patterns and mechanisms involved, such as the discontinuity of the process of evolution across the prokaryote-eukaryote divide, summons forth a pluralistic approach to studying evolution.

**Reviewers:**

This article was reviewed by Ford Doolittle, John Logsdon and Nicolas Galtier.

## Background

### The history of life and the tree of life: How similar are they?

Even before Darwin, biologists used the metaphor of a tree to classify living things [1]. The most prominent historical example, however, is indeed Darwin's "great tree", which extrapolated a family genealogy to the level of species and beyond to describe the historical relationships between living entities. He wrote:

*The affinities of all the beings of the same class have sometimes been represented by a great tree. I believe this simile largely speaks the truth. The green and budding twigs may represent existing species; and those produced during each former year may represent the long succession of extinct species. .... The limbs divided into great branches, and these into lesser and lesser branches, were themselves once, when the tree was small, budding twigs; and this connexion of the former and present buds by ramifying branches may well represent the classification of all extinct and living species in groups subordinate to groups (1859: 120) *[2].

This image of a tree has resonated cognitively and visually with both biologists and the wider public, and the history of systematics attests to an increasingly popular goal of classifying all organisms not just evolutionarily but also within a unique and ever-bifurcating pattern of representation, a grand natural system in the shape of a tree. Such a representation of life's history is now widely known as the 'tree of life', often capitalized, with modern manifestations including all of the prokaryotes and the protists (eukaryotic microbes). The tree metaphor, while a helpful descriptor for the genealogical relationships of macroscopic life, does not describe prokaryote evolution over the vastness of evolutionary time.

Darwin's theory of descent with modification operates with just two mechanisms, natural variation (or heritable variation) and natural selection, acting over vast spans of geological time. The theory was formulated largely on the basis of observations of multicellular eukaryotes, organisms visible to the naked eye. Importantly for our arguments here, the tree metaphor came to be connected with the theory of evolution at a time before biologists had any ideas about the mechanisms underlying the principle of natural variation. Although our concepts about the workings of natural selection are hardly different today than Darwin's 150 years ago, our modern understanding of the mechanisms of natural variation are orders of magnitude more detailed than anything Darwin could have imagined. Furthermore, and of central importance to our case, we now know that the mechanisms of natural variation are not uniform across all forms of life. Rather, there is a discontinuity of evolutionary mechanisms, in particular and most importantly across the prokaryote-eukaryote divide.

At the level of cellular organization the deepest divide in the living world is that separating eukaryotes from prokaryotes [3-5]. The same is true that when we view the world from the standpoint of mechanisms underlying natural variation. It is undisputed that the genetic mechanisms that generate point mutation and chromosome replication errors are common to both prokaryotes and eukaryotes; it is also undisputed that mutations inherited via chromosome replication and cell division give rise to tree-like structures over time. But the mechanisms of natural variation entailing recombination in its various forms differ starkly between prokaryotes and eukaryotes. Among eukaryotes, meiosis ensures reciprocal recombination among homologous chromosomes and reassortment of alleles within lineages that recombine within or very near (in the case of hybridization) species boundaries (see [6] for more details). Moreover, the basic molecular machinery of meiotic recombination and sex was present in the eukaryote common ancestor, as recent studies strongly suggest [7-9]. Over geological time that process, which includes chromosome replication errors, generates tree-like structures during eukaryote evolution. While lineage sorting [10], introgression [10,11], and endosymbiotic gene transfer [12] are important deviations from a strictly bifurcating process, no one would doubt that vertebrate evolution can be approximated by a tree, with furcations, lineages splittings and no significant amount of reticulation between divergent lineages.

But in prokaryotes, the mechanisms of natural variation are quantitatively, and many would say fundamentally, different from what goes on in sexual eukaryotes. These mechanisms include transformation, transduction [13], conjugation [13], gene transfer agents [14] and integrons [15-17]. For instance, recent findings at Lost City hydrothermal field, a specialist environment with low organismal diversity among prokaryotes but a tenfold higher frequency of transposases than any environment studied before [18], indicate that transposase-mediated LGT is a significant and selected contributor to genetic diversity in that environment. In the context of a donor and a recipient cell, these processes of recombination are always unidirectional, never reciprocal; they can cross taxonomic boundaries; and they entail the movements of fragments of chromosomes rather than whole chromosomes. Operating over geological time scales, they result in observations among prokaryotes of the following sorts:

• The new species, *Nautilia profundicola sp. nov*., recently discovered in deep-sea hydrothermal vents and assigned to the genus *Nautilia*, shares only 35% of its DNA sequences with the previously characterized species of *Nautilia *[19].

Three individuals among the same "species" of *Escherichia coli *may typically share only 40% of their combined set of genes. By contrast, three individuals of the same species of eukaryotes generally have a nearly identical gene repertoire.

• Strains of the marine heterotrophic bacteria *Vibrio*, which are identical at one or more protein-coding housekeeping loci, can be highly differentiated in genome size (up to 800 kb variation, ~20% of the genome) [20]. Likewise, strains of the nitrogen-fixing soil bacteria *Frankia *with more than 97% identity in their rRNA sequences (considered to be the same species under most traditional definitions) can differ by as many as 3500 genes. This number represents at least 43% of the genes of the larger genome of these 3 strains, and up to 77% of the genes of the smaller genome of *Frankia *[21].

• Comparison of the genomes of pathogenic and symbiotic bacterial strains with their close free-living relatives shows that pathogenicity islands and similar symbiosis islands, clearly acquired via lateral gene transfer (LGT or HGT), can comprise over 30% of a bacterial genome [22-24].

• A number of phylogenetic analyses show that although the rate of LGT between divergent organisms might be lower among highly conserved genes involved in information processing than it is among metabolic enzymes and other "operational" genes [25], multiple LGT exists at the very heart of the translation system [26-28]. Informational transfers also occur among ribosomal protein genes and rRNA encoding operons, transcription system genes, and RNA polymerase subunits [29].

We have become accustomed to hearing such examples of extensive chimerism and lateral gene transfer among prokaryotes, as if they were common-place. They are. There are no comparable observations among multicellular eukaryotes that would even approach this degree of massive chimerism, notwithstanding the endosymbiotic origins of chloroplasts and mitochondria and their associated gene transfers from organelles. The reason is that the mechanisms of natural variation are different across the prokaryote-eukaryote divide. Processes that deviate from the strictly bifurcation pattern of descent also occur among eukaryotes, including multicellular plants and animals [30]. Yet, the extent of chimerism among prokaryotes is much more dominant, because it is at the core of processes generating natural variation in these groups.

The mechanisms of unidirectional spread of genes among prokaryotes may be slow at the level of individual generations, but over geological timescales, their cumulative effects are vast. How vast? Although the degree of the effects that lateral gene transfer has is thought to be highly variable across prokaryotic lineages [31,32,15], the bottom line of any debate on this issue is that it is entirely plausible that LGT has affected every single gene in prokaryotes over the full span of evolutionary history depicted by the tree of life. If we recapitulate the development of evolutionary thinking from Darwin through the Modern Synthesis into the age of genomes, we see that genomes have provided major bodies of evidence for the prevalence of vertical descent among multicellular eukaryotes, with sexual recombination, mutation, gene and genome duplications being the main processes that give rise to genetic novelty. At the same time, genomes gave microbiologists evidence just as pervasive for the workings of lateral gene transfer during prokaryotic evolution. However, we have taken the belief in a fundamentally tree-like process of evolution as observed among most multicellular eukaryotes and by extrapolation have projected it onto the evolutionary history of prokaryotes. To sum up our arguments thus far in a single sentence: **The belief in the existence of a universal tree of life - inclusive of prokaryotes - is stronger than the evidence from genomes to support it**.

We are emphasizing the prokaryote/eukaryote dichotomy, and the discontinuity of evolutionary process across that divide, in order to develop the point that although the principle of natural variation is uniform across all life, the processes and mechanisms underlying it are not. This discontinuity raises questions as to how, whether, and to what extent we can view prokaryote evolution through the image of bifurcating trees without obtaining a wholly distorted picture of the process. Either we have one evolutionary mechanism and one evolutionary model that applies to all life, hence one tree of life, or we have a plurality of processes and models in particular to accommodate the differences between prokaryote and eukaryote evolution. It is important to keep in mind nonetheless that, although prokaryotes and eukaryotes evolve in very distinct ways, justifying in our view different evolutionary models for their study, most of the protistan diversity remains currently unexplored and unsequenced. We acknowledge that it is already known that in single-celled eukaryotes, endosymbiosis and gene transfer are important processes for innovation [33-37], and the extent to which evolution of some protists can be approximated by a furcating tree is currently debated. Increasing knowledge of the genomes of protists may thus in the future expand our conclusion: not only are the tree of life and prokaryotic evolution are two different things, but all microbial evolution (that of prokaryotes and protists) may also be poorly described if addressed in an exclusively tree-like framework.

In the following we will consider this circumstance from philosophical, scientific, and epistemological perspectives, surmising that phylogeny opted for a single model of evolution due to the dominance of the Modern Synthesis account of evolution (which was largely prokaryote-free). We will argue that the universal tree of life, the single traditional model to describe evolution, is no longer the most scientifically productive position to hold. Forcing a single bifurcating scheme onto prokaryotic evolution disregards the non-tree-like nature of natural variation among prokaryotes and accounts for only a minority of observations from genomes. Hence we will briefly set out alternative models to the tree of life to study their evolution.

## Results and Discussion

### One model (monism) or many (pluralism) to study evolution?

Philosophers have often debated whether one model or many ought to be used in science, identifying schematically two positions among scientists: the monists and the pluralists. Those who are inclined to use a single model to account for all of their data, however complex these might be, are traditionally called monists. The remainder are pluralists. In its simplest description, monism designates a commitment to one model to which all other evidence and interpretations must be subordinated [38,39]. For instance, in physics, monism is justified by the appeal for a single system of fundamental laws that could explain all physical phenomena. Searching for a unified explanation is seen as the essence of good scientific practice, since in that context hypotheses are rigorously applied, evidence interpreted consistently, and all findings coherently unified by fundamental principles into one overarching theoretical framework. In evolutionary biology, this approach would be relevant, for instance, if evolution were a thoroughly homogeneous, structurally simple process. Then it might be that we should look at the understanding of evolution as, in effect, a single question, calling for a single mode of analysis. And this is, essentially, the assumption implicit in much neo-Darwinist thinking. Furthermore, monism comes in general with an ontological commitment to a particular class of entities as the organizing theoretical focus. Typically, in the case of traditional phylogenetics, these chief objects of study would be the species. Whether species history is being traced by genes, genome composition or something else, the traditional role of phylogeny is to recover their relationships. Consistent with that approach, traditional phylogeneticists consider that species evolution follows a tree, and processes such as LGT are theorized as supplementary and thus unthreatening. From that standpoint, even if all individual prokaryotic gene trees disagree, there is still some universal species tree. In that sense, it seems that scientists exclusively committed to the reconstruction of one single universal tree (the tree of species or tree of life) embrace or are inspired by a monistic perspective on the process of evolution, in which lateral processes are not admitted at all or play only a secondary role. In the rest of this manuscript, we will call this position tree-monism.

However, there are objections to a monistic approach, and not only in biology. Pluralism opposes monism. Pluralism in philosophy of science (and political philosophy) means the conviction that different models may be legitimate to analyze a phenomenon, and that conflict between them need not be seen as invalidating one or more alternative approaches [38,39]. Many pluralists would justify their pluralism with the claim that the world itself is not carved up in a way that is conducive to the application of one approach only, and that a richer understanding of the phenomena can be gained with the application of more than one approach. Pluralism should be unsurprising for biologists since they are dealing with thoroughly complex objects. Thus their scientific models, to provide any possibility of insight and understanding, must focus only on specific and limited aspects of this complex reality. One should then anticipate that different questions should best be addressed using different concepts or models. This has important bearing on our practice of evolutionary biology. Once it is accepted that different classes of biological entities are evolving to some extent in different ways (as do prokaryotes and eukaryotes, for instance), then it is a wholly empirical question to what extent the same processes will be equally significant in explaining evolutionary histories. It is also an entirely empirical question whether the perspective best fitted to gaining insight into one class of objects or processes (e.g. the eukaryotes) will be the same as that most appropriate to another (e.g. the prokaryotes) and, indeed, whether any single perspective will adequately illuminate a particular class of objects or processes. With regard to the tree of life, the pluralistic position has thus been regularly advanced by microbial phylogeneticists who have emphasized the diversity of evolutionary processes and entities at play in the microbial world [40,41]. This group prefers to model evolution as a diverse set of processes acting on the histories of diverse kinds of entities generating, finally, a diversity of overlapping and cross-cutting patterns, corresponding to different evolutionary outcomes. For such pluralists, depending on the approach taken (e.g., the choice of sequence, the choice of the reconstruction method, the taxa of interest), a different evolutionary pattern may be generated (e.g. a reticulated network rather than a vertical tree). Embracing this latter view, we will now argue that using a single tree-like model to describe all life evolution is no longer the most scientifically productive to hold. In other words, we should approach the study of prokaryote genome evolution openly, and no longer subordinate our approaches to the study of microbial evolution to the preconceived notion of a tree.

### Limits of traditional tree-monism

In addition to its limits in accounting for the different evolutionary processes emphazised by the prokaryote/eukaryote divide, there are many methodological and epistemological reasons why tree-monism may not be any longer the most scientifically fruitful position from which to study microbial evolution. We will examine some of these issues in order to show how tree-monism falls short in many ways.

#### Methodological issues

##### Problem 1: The circularity and arbitrariness of tree methods

The most traditional tree of life hypothesis, ignoring LGT, predicts that trees of single-copy genes (orthologs) from a common taxonomical sampling should be congruent with one another and with the species tree. Thus, the goal of the phylogenetic analysis has long been to reconstruct this common topology. No gene tree alone can fully resolve the entire species tree of all life forms [31], so genes are often combined into a single analysis under the tree-monistic assumption that they all share the same vertical history. In doing so, the aim is to reduce effects of small sample size (stochastic errors) in phylogenetic calculations, thereby reinforcing the true phylogenetic signal [42,43]. Unfortunately for this assumption, LGT means that there is no *a priori *guarantee that a common tree is really present in the molecular data. Worse, it is currently not possible to provide positive evidence that the roughly three dozen genes that have been claimed to save the concept of a universally shared core from extinction [44,45] actually do share a common history [46]. Hence, there is a high risk that the traditional approach produces circular phylogenetic analyses, in which assumptions of a common tree are supported by assumptions about how the data should be represented. As noted by Avise, "*any comparative dataset can be used to reconstruct a phylogenetic tree when a tree provides the suppositional metaphor for the data analysis. Even inanimate entities (such as different kinds of chairs or cars) can be grouped into tree-like depictions based on their similarities or differences" *[47]. A typical example of such an arbitrary tree is Cicarelli et al.'s tree of life [45], which is based on 34 concatenated orthologs. When tree assumptions are removed, their data reveals a great deal of LGT and many genes whose history is simply unknown [46].

##### Problem 2: Underestimation of phylogenetic incongruence; exaggeration of congruence

To avoid the arbitrary issues associated with combining genes into a single tree, statistical tests attempt to examine whether different gene-tree topologies could be due to chance [48]. In those tests (e.g., character congruence tests such as the incongruence length difference test [49] and variants, or likelihood based tests), the null hypothesis (H0) is "*that the same tree underlies all of the dataset partitions*" [48]. The alternative hypothesis, H1, proposes that some of the genes being compared have undergone a different history. It is then statistically incorrect to say that when "*genes do not significantly reject the consensus tree*" (H0), that "*agreement seems to be the rule*" [50]. First, in purely statistical terms, this failure to reject does not mean that they support the consensus tree, and that they have evolved according to this very topology [51]. Second, individual genes with a weak phylogenetic signal will always fail to reject the consensus tree.

Fortunately, the critical power (and relevance) of such simple congruence tests can be illustrated by studying an increasing number of independent test topologies, "supported" or "rejected" by individual genes. To do this, the Shimodaira-Hasegawa or Approximately Unbiased tests [52,53], which hold the null hypothesis that all the trees tested are equally good explanations of the data (and the H1 hypothesis that some trees are better explanation of the data), can be used [48]. In particular, testing independent topologies leads to the identification of genes that simultaneously fail to reject many different trees. If the failure to reject one tree meant straightforwardly that this tree should be accepted as representing the true phylogenetic history, then one would have to assume that a gene simultaneously failing to reject multiple incompatible topologies evolved to produce many incompatible phylogenetic histories. A more realistic explanation is that such a gene contains too weak a phylogenetic signal, given the assumed substitution model, to decide what its history was.

Shi and Falkwoski's work illustrates one approach of how to critically study genes with a weak phylogenetic signal, without claiming that data are congruent with one tree when there is no genuine support for it [54]: First, they built phylogenetic trees for 682 orthologous protein families from 13 cyanobacterial genomes and did not observe any predominant, unanimous topology that represents a large number of orthologs. The maximum number of orthologs that share a particular topology accounts for only 1.9-2.1% of the orthologous datasets [54]. Then, they reconstructed five test topologies: the consensus tree, the ML and NJ supertrees, and the ML and NJ concatenated trees for these alignments. They observed that almost all (97.5 to 99.6%) of the molecular datasets supported the five topologies at the 95% confidence level, suggesting a lack of resolution of single gene phylogenies. Had they only tested the agreement of the individual gene phylogenies against one of these five candidate trees of cyanobacteria, they could have mistakenly concluded that they had found The Tree of Cyanobacteria.

##### Problem 3: Large-scale exclusion of conflicting data

Methods that search for a single universal tree often involve steps of data exclusion in which lateral gene transfer is conceived as noise. The use of such eliminative criteria allows these phylogeneticists to ignore LGT, but also leaves them without any trustworthy genes with which to study prokaryote evolution. Soria-Carrasco and Castresana's "Estimation of phylogenetic inconsistencies in the three domains of life" [55] is a good example of this logic. These authors compared the level of incongruence in proteobacterial genes and eukaryotic genes to test whether the proportion of vertical/lateral signal significantly varied between these taxa. They argued that if these levels were comparable between eukaryotes and proteobacteria, LGT could not be considered a major evolutionary process in these bacteria. Through recurring steps of data exclusion, they removed as much conflicting data as possible to guarantee that no phylogenetic difference could be found between the eukaryotic and proteobacterial data.

First, they retained only ubiquitous "core" genes, thus throwing out of the analysis the majority of the prokaryotic data in order to avoid taxonomical patchiness. The disagreement between these individual "core" gene trees and the "species tree" (i.e. the concatenated gene tree) was, however, higher for prokaryotes than for eukaryotes. Consequently, in a second step, the authors excluded all genes for which there was more than one copy per species. The aim was to exclude duplicated genes both from the eukaryotic and prokaryotic datasets, due to a suspicion that the large amount of incongruence observed in bacteria could be due to excessive duplications and losses. Yet, such a procedure obviously excluded the paralogs as well as any multiple copies resulting from lateral gene transfers in prokaryote genomes. Only 127 genes could be retained for proteobacteria, as opposed to 346 for eukaryotes.

Nevertheless, prokaryotic gene trees continued to show more disagreement with the concatenated gene tree than eukaryotic genes did, and this prompted a third exclusion step. Biases in gene length were corrected, since proteobacterial sequences were smaller on average than eukaryotic sequences (214 aa versus 251 aa). All genes were trimmed to an identical length of 182 unambigously aligned positions. Based on this reduced dataset, the AU test indicated that 46.5% of the individual proteobacterial genes were incompatible with the "species tree" as opposed to only 23.4% of the eukaryotic alignments. The authors then dismissed these results by arguing that the gene lengths were now too short to conclude anything about the impact of LGT. So, in a final step of "good" gene selection, they removed all markers shorter than 300 aa and retained only 88 eukaryotic genes and 20 proteobacterial ones for their comparative analysis. But even in this heavily curated dataset, the AU test demonstrated a higher level of incongruence within the proteobacterial dataset (25% incongruence) than within the eukaryotic dataset (14.8% incongruence).

Even though the "purified" data now amounted to a mere 0.8% of the size of a bacterial genome, and are obviously unrepresentative of the evolution of the rest of the proteobacterial genome, the authors surprisingly concluded that overall no more LGT could be observed in proteobacteria than in Eukaryotes. According to them, such a study "*opens the way to obtain the tree of life of bacterial and archaeal species using genomic data and the concatenation of adequate genes, in the same way as it is usually done in eukaryotes*." [55] From a pluralistic point of view, however, it is striking that a great majority of the bacterial data have to be excluded to achieve the reconstruction of a so-called "universal" tree. In other words, almost none of the data that Soria-Carrasco and Castresana examined fit the metaphor of a tree, but they nonetheless filtered their observations to sieve out only those that were compatible with their preconceived notion that the evolutionary process is tree-like in both groups. The result is that this forced them to disregard most of the data they initially wished to explain evolutionarily.

##### Problem 4: Deprioritizing conflicting data

For those who take a monistic approach, sidelining or deprioritizing data that conflicts with the model of a single tree may appear to be a less extreme alternative than large-scale data exclusion. One such example is Daubin and Galtier's recent proposal to build a tree of life by dismissing the plethora of incongruences in molecular data. For them, "*the existence of incongruences is not sufficient to dismiss the notion of a species tree, nor to preclude its reconstruction*. [...] *In our view, the species tree could still be a useful concept even if incongruent with every gene tree*" [50]. They argued that from a statistical point of view, rejecting the species tree because of the existence of conflicts between gene trees means refusing to calculate the mean of a distribution because its variance is non-zero, which appears too extreme a policy [50]. They claim that the species tree can be recovered even when the variance in phylogenetic signal is extensive, as long as transfers occur randomly. Furthermore, they assert that one could interpret the mean and variance in phylogenomics differently: the mean signal corresponding to speciations/extinctions, and the variance to LGT and other non-vertical processes [50].

Daubin and Galtier are suggesting that calculations of the mean phylogenetic signal of incongruent genes are the best way to build a tree of life because it integrates (in reality, averages) a large amount of incongruent data. Under their assumptions, "*a supertree method (which essentially returns the "average" estimated gene tree) recovers the true species tree with strong accuracy from phylogenomic data simulated under a model incorporating LGT, even when the amount of LGT is such that two random gene trees share only 50% of their internal branches, on average*" [50]. Although it is curious that anyone would summarize such a reticulated pattern with a tree, a deeper problem with such claims is that lateral gene transfer does not in fact occur randomly. It is strongly influenced by the selective processes operating in organismal environments.

For example, the bacteria *Salinibacter ruber *displays many genes linked to adaptation for life in hypersaline environments. These genes have their closest homologs in the genomes of co-habitating halophilic archaea [56]. A similar example can be found in the archaeal genera *Sulfolobus *and *Thermoplasma*. Despite belonging to different phyla, 17% of their genes are each other's closest homologs [57]. This mutuality can be explained by extensive lateral gene transfer between these organisms, as they evolve to thrive in the same types of environments (high temperature and low pH). Furthermore, vertical and lateral evolutionary signals are entangled with one another in molecules, such that it becomes difficult to distinguish them through simple tree-centred approaches. If we really want to understand evolutionary process and pattern, it seems clear that simply deprioritizing lateral signal will be a mistake.

##### Problem 5: Ambiguities in tree of life patterns

Several observations question the validity of equating the consensus or average phylogenetic pattern with a bifurcating evolutionary organismal history, or with the tree-like evolutionary history of the species [58-61]. At least some of the consensus signal found in core genomes [60] might reflect not a shared history but instead, artefactual phylogenetic reconstruction. Many phylogenomic studies have produced a "reference tree" that is an aggregate constructed from many individual genes. Using 16S rDNA trees as an explicit or implicit comparative criterion, these aggregate trees have been claimed [45] or used in practice [62-64] as a vertical scaffold onto which LGT events can be mapped. Whether constructed using a supermatrix, supertree or other approaches, it is often possible (and always desirable) to attach estimates of statistical significance to features of such aggregate trees. Supermatrix-derived phylogenies can be subjected to bootstrap or jackknife analyses in the same manner as single-gene phylogenies, while other approaches such as supertrees can be resampled using techniques that are appropriate to the underlying data, e.g., bipartitions in a supertree constructed using the Matrix Representation with Parsimony [65,66] method, and other support indices [67].

Such measures of statistical support can be extremely misleading, however. It is widely known, for instance, that support values such as the bootstrap proportion or posterior probability can strongly support an incorrect split in a tree due to model violations or multiple phylogenetic histories within a data set [68]. It is therefore necessary to test whether strong support for a given split in an aggregate tree is found consistently in all or a majority of the contributing entities (i.e., single-gene alignments or individual phylogenetic trees). In one such supertree [64], a sister relationship between *Aquifex aeolicus *and *Thermotoga maritima *was reconstructed as the earliest-diverging group within the bacterial supertree. A total of 120 trees in the input data set yielded a 'strong conclusion' about this relationship, either resolving *A. aeolicus *and *T. maritima *as sisters with strong Bayesian posterior support (PP ≥ 0.95), or displaying an alternative relationship in which the two were placed with other partners, again with strong support. Only 20 out of the 120 trees supported the pairing of these two taxa. Furthermore, analysis of alternative relationships showed many distinct partners for *A. aeolicus*, including several branches within the Proteobacteria, as well as both the Euryarchaeotes and the Crenarchaeotes, and the genus *Clostridium *(which includes the thermophile *Thermoanaerobacter tencongensis*). Similarly, *T. maritima *showed strong affinities for several Gram-positive lineages (especially *T. tengcongensis*), *Pyrococcus *and *Chlorobium tepidum*. While more than 30 trees supported a relationship between *Aquifex *and basal Proteobacteria, the *A. aeolicus*/*T. maritima *pairing was nonetheless favoured by the MRP algorithm. It is thus highly debatable as to whether this latter relation should be considered as the true vertical signal.

Another example can be found in analyses of *Thermoplasma*, which is a genus of hyperthermophilic euryarchaeotes that often branches near the base of the Archaea in aggregate trees [69,70]. However, concatenated informational protein phylogeny[71] places *Thermoplasma *within the euryarchaeal methanogens. Analysis of the quartet relationships between *Thermoplasma acidophilum*, the euryarchaeotes *Methanopyrus kandleri *and *Pyrococcus horikoshii*, and the thermoacidophilic crenarchaeote *Sulfolobus tokodaii *from the Beiko et al. (2005) [64] dataset yielded 22 quartets that placed *T. acidophilum *with *S. tokodaii*, consistent with the reference supertree. 22 other quartets supported a sister relationship between *T. acidophilum *and *M. kandleri *(consistent with the informational protein phylogenies of another study [71]), and another 21 supported *T. acidophilum *with *P. horikoshii*. Quartet analyses with *T. acidophilum *and other triplets of genomes yielded relatively weak support for the basal positioning of *Thermoplasma *in the Archaeal part of the supertree. Instead, two alternative placements within the Euryarchaeota and Crenarchaeota were supported. Furthermore, it is noteworthy that most supertree methods can produce novel clades not supported by any of the source trees [72].

Even though simulated random LGT regimes tended to diminish statistical support for more-ancient relationships rather than offering strongly supported alternatives in average trees, phylogenetic approaches have been shown, in theory and in practice, to favour one topology even if the input data are generated equally on two or more trees [73,74]. Compositional or rate effects may be sufficient to give strong statistical support to a grouping of branches that should in fact be unresolved [75]. Indeed, systematic biases in residue composition have been shown to influence large, concatenated phylogenies such as those of eight species of yeast [76]. Likewise, most phylogenetic reconstructions methods to-date assume a time-reversible model, while compositional bias in fact changes during evolution. The assumptions of this model are thus frequently violated, especially if different genera, families, or even phyla are included in the same reconstruction. Likewise, when data are simulated under biased regimes of LGT and a genome phylogeny approach, the recovered tree displays neither the complete vertical history, nor that of any significant pathway of LGT [77].

Furthermore, gene transfer can create patterns indistinguishable from those created by vertical inheritance, as was first recognized when the extent of gene transfer among bacteria became visible in comparative genome analyses [78-80]. It is reasonable to assume that the rate of successful transfers relates to overall similarity (use of the same transfer machinery, phages that infect both organism, similar machineries for transcription and translation, and similar signals functioning in replication and genome organization [81]). Gene transfer biased towards similar partners reinforces the similarity that leads to more gene transfer. The transfers thus create a signal that groups organisms together, such that we consider them to be closely related. In some instances these gene transfers might reinforce a signal due to shared ancestry, but in other instances all of the signal that we detect today might have been created by gene transfer itself. The claim that the consensus tree recovered in some molecular phylogenies is based on shared ancestry hitherto remains an unproven assumption. What remains are two processes, vertical inheritance and gene transfer, both of which contribute to recovered trees in ways that can be difficult to distinguish using only one model.

Consequently, any statistically well-supported tree recovered from a phylogenomic analysis should not be construed uncritically as a 'tree of life' unless hybrid signals and model violation effects are considered and rejected as potential confounding factors.

#### Epistemological issues

Beyond these methodological issues, adherence to the traditional tree of life raises substantial epistemological issues, about the very nature of the knowledge generated.

##### Problem 6: What are trees of life really trees of?

As discussed above, the simplest tree-of-life rescue strategy currently used is to group some genes, including those which might have different histories, and calculate the "average" tree-like history of these genes [50]. The analyst lumps together a great deal of data that did not evolve by a common tree-like process, analyzes it with methods that deliver only trees as their result (as opposed to more-general models such as networks), obtains a tree, and then asserts that this exercise provides evidence in favour of the existence of a tree. A second tree-rescue strategy is to select some smaller set of "core" genes and come up with a tree based on their divergence. A final tree-rescue strategy is to view a "variable core" as defining the tree of life. Known as supertrees, these trees do not represent the histories of even a small set of genes, but instead reflect the inheritance of different genes at different nodes [82]. What these strategies have in common is a commitment to uncovering tree-like inheritance patterns in the complexity of microbial inheritance. The question is whether they really do result in a hierarchy that corresponds to the tree of species, or whether they are in fact teaching us something else altogether about prokaryote evolution.

Consider first the averaging strategy. A species is composed of organisms, and those organisms are composed of parts whose histories differ. Some genes might have been transmitted "vertically" through much of their histories, while others might have been transferred from closely or distantly related taxa at various past times. If we average these histories, what does the resulting tree represent? The simple problem is that the historical branch-points on such a tree do not necessarily represent past species. We don't have a species history here at all. Even Galtier and Daubin admit that not even a single gene might have followed the path represented by the average tree. No real species would necessarily correspond to these averages. Averaging the tree signal would be akin to asking about the 'geographic average' destination of an American business traveller, which would probably be (i) somewhere in Iowa, and (ii) would not convey much meaningful information. Such a central tendency tree should thus be critically interpreted by biologists, and not conflated with the universal species tree.

In the second tree-rescue strategy, the search for a core, a scientist attempts to separate the wheat (vertically transferred genes) from the chaff (genes that underwent LGT). Such methods do, of course, yield tree-representations. Proponents thus claim that if there is such a set of core genes, "a tree of bacterial species remains possible" [58]. Yet, the main difficulty with the claim that the history of the core genes represents the species history is that all we can safely conclude from the history of the core genes is simply knowledge of the history of the core genes. A species--and the organisms that comprise it--have histories that are not exhaustively explained by the histories of a few of their parts. To maintain that the history of the core genes "represents" the species history requires some argument that the history of these parts is somehow "essential" to a species' genealogy. But post-Darwinian biologists are generally loath to attribute any special essentialist status to either genes or species. If they fail to essentialize (which should be expected), then any such core-gene tree, which might well be an interesting and at times scientifically fruitful representation, cannot be considered to represent the *species *history.

Finally, in the supertree strategy, the transmission of individual genes is not used to create a tree-scaffold, but instead different genes in different parts of the tree of life are combined. More precisely, different markers, presenting very little overlap in their taxonomical samplings, are used to reconstruct different parts of the tree. It is assumed they all fit on a common tree, despite the fact that there is little or none support in such a patchwork of data for many inner nodes. This strategy can appear to increase the size of the core, since the genes that persist across a speciation event, or even a series of such events, will not be whittled away merely because those same genes are transferred in some other part of the tree. But does this strategy represent a species tree? Again, the problem is one of representation. There is certainly some pattern in nature which answers to this description. Perhaps a supertree representation accurately reflects the history of cell division. However, to call this a species tree is to claim that all important species characteristics are inherited along these lines - a claim that is exceedingly hard to justify.

Because none of the options described above accurately reflects species trees, we should instead strive to describe prokaryote evolution as it is in nature. That may require a departure from analytical methods that only operate in the language and mathematics of trees. Networks, for example, offer an alternative mathematical framework, albeit one that is not necessarily compatible with a tree-monistic concept of inheritance or speciation.

##### Problem 7: Tree monism no longer provides the ideal comparative evolutionary framework

In the time before genome sequences, when there was *bona fide *reason to "hope" that prokaryote genomes would uncover vast evidence for common ancestry, the goal of obtaining a universal tree of life promised to serve three highly desirable purposes. First, it would provide a natural classification of living organisms, by identifying all the extant descendants of a given ancestor forming a natural group. Knowing the tree of life would thus conveniently define a hierarchical classification of Life, the "groups within groups" proposed by Darwin. Second, this tree could provide insights into the shared properties of organisms belonging to the same group, and allow generalizations about the natural groups. Third, this tree could be seen as a time machine. Knowing its topology, and the properties of the extant organisms, to a certain extent one could infer the properties of the ancestors (i.e. achieving retrodiction) by assigning properties that are common among all descendants to ancestral nodes. For all these reasons, the universal tree seemed the best possible comparative framework for evolutionary biology, and ribosomal RNA was occasionally referred to as "the ultimate chronometer" [83].

Today however, if embracing a monist perspective to describe microbial evolution, the question is not to ask *whether *the tree model still represents the best framework to infer and depict evolutionary relationships, but rather to ask *which *of the competing approaches already available is best suited to produce the most satisfactory tree. A wide array of methods have been developed not only to address LGT, but also to deal with gene conversion, recombination or hybridization (for reviews, see [84-88]). All of these so-called reticulation events are the product of various biological processes that violate the universal tree model. Consequently, they directly challenge its utility for classification, generalization, and for retrodiction, since any attempt to treat evolution as a tree-like process is insufficient even if partially useful [30].

Consider the analogy of the origins of organelles via endosymbiosis in eukaryote evolution. It vividly demonstrates that the notion of a generalized tree of life is not the most productive position to hold. It highlights an important manifestation of the discrepancy that arises between hierarchical classification using the structure of a tree on the one hand and evolutionary process on the other hand, when the evolutionary process is not tree-like to begin with. Plastids arose from cyanobacteria, and mitochondria (including their anaerobic and non-ATP-producing forms, hydrogenosomes and mitosomes) from proteobacteria. Both organelle types (usually) still possess their own genome, and both symbioses entailed gene transfers from those endosymbionts to the nucleus during the evolutionary transition in which those endosymbionts became organelles [36,89]. Moreover, some current views have it that the origin of mitochondria was contemporaneous with the origin of eukaryotes themselves [90-92], that the host for the origin of mitochondria stems from within the archaebacteria [93], and that the origin of photosynthetic eukaryotes was contemporaneous with the origin of plastids [35,94]. Although there are still some controversies around this scenario, the main point is that the endosymbiotic origin of plastids and mitochondria does not conform to the tree paradigm. Both eukaryotes in general and plants in particular represent genetic mergers in evolution, cellular marriages consummated by the genetic integration afforded by endosymbiotic gene transfer and protein import by organelles.

Thus, any tree of life that makes the effort to link prokaryotes and eukaryotes in a manner that reflects the underlying evolutionary process would need to include archaebacterial-eubacterial lineage mergers at the origin of mitochondria/eukaryotes and eukaryote-cyanobacterial mergers at the origin of plants. Similar mergers occur in the origin of algae that possess secondary plastids [95]. But if we force the metaphor of a bifurcating (or multifurcating) tree onto the evolutionary process linking prokaryotes and eukaryotes, then we have to decide whether to put the eukaryotes on the host lineage or on the mitochondrial lineage, and we have to decide whether to put the plants on the cyanobacterial lineage or on the eukaryote lineage, when in fact the endosymbiotic origin of these organelles ends up putting the resulting organisms on both branches at once.

The discrepancy is even greater between a hierarchical classification of prokaryotes and lateral evolutionary processes. When Cicarelli et al.[45] attempted to identify (by hand, ultimately, even though the paper advertised an automated method in the title) all the genes that had not been lost or transferred among genomes representative of all life, they ended up with 31 genes, corresponding to about 1% of the genes in a typical prokaryote genome. The authors assumed that those genes tended to produce congruent trees, rather than demonstrating that they actually do. In other words, at face value they found that about 1% of any genome at best might tend to fit the working hypothesis of a tree. Any reasonable account of scientific method would suggest that when a working hypothesis can only account for about 1% of the data, a true scientist would start looking for a better working hypothesis. The current retention by many evolutionary biologists of a strict tree metaphor for prokaryotes, despite its inability to account for the observations, presents a serious barrier to our understanding of prokaryotic evolution and is hard to square with most accounts of how science should be done.

On the other hand, despite their differences, all the evolutionary processes listed above can be modelled and represented simultaneously by phylogenetic networks better than by trees, if a unique representation is desired. It thus seems both prudent and pragmatic to explore alternative mathematical representations of microbial evolution. Adoption of network strategies does not constitute rejection of significant bifurcating patterns in the history of life. Instead, it requires the denial that tree-patterns are the *only *possible patterns. Leaving aside the specific methods to detect LGT [69,96], recombination [97], gene conversion [98], hybridization [99] and other reticulation events [100], different algorithms have now been proposed to build phylogenetic networks or to represent the non-tree component, such as weak hierarchies, split decomposition, netting, statistical parsimony, minimum spanning networks, reticulograms, median networks, median-joining networks, union of parsimony trees, and neighbor-net [101-109]. Consensus methods for assembling incompatible trees into networks and supernetworks are also available [110].

In light of all these approaches, algorithms and software already published (and still being developed), the search for optimal trees could be advantageously replaced by the search for optimal networks. Because trees are special types of networks, the tree model is most properly understood as embedded in the network-model of evolution [111]. The paradigmatic shift from a monistic to a pluralistic understanding of the evolutionary processes is thus echoed by a graph-theoretical shift, from trees (i.e, connected acyclic graphs) to networks (i.e., connected graphs which may contain reticulations). Indeed a good network approach will always return a tree if the underlying data have a tree-like structure (for distance data, the four-point condition has to be satisfied). However, if significant conflicting signals are present in a data set, then suitable network methods should be able to depict reticulation events that a strictly tree-based approach cannot. Although network methods have limitations [112], they should nonetheless permit progress towards more accurate representations of the process of microbial evolution as it occurs in nature, as opposed to depicting how some of us think it might occur by extrapolation from observations and experience in the study of vertebrates.

With so many methods available, the real problem is to assess the relative performance of the competing approaches with simulated data [77,86,113-115] as well as in real-case applications [116]. The problem of identifying the minimum number of reticulations in a graph is NP-hard [117], such that most recent developments in this field have been to develop good algorithms to approximate the optimal solution [118,119]. If it is accepted that networks are the best model to study LGT and microbial evolution, the next problems arise of how to assess the likelihood [120] and robustness of such networks [121], and to compare networks or determine when a network is significantly more informative than a tree [122]. Although methodological and algorithmic limitations may have precluded the use of phylogenetic networks in the past, a few steps have been taken in this direction [123]. It is time to show much more of the evolutionary process.

### Process pluralism and its implications for taxonomy

Many of the above limitations associated with a tree-monistic approach in reconstructing the tree of life could easily be dealt with by assuming a more pluralistic approach to describe microbial evolution. We already know that microbial evolution and the tree of life are distinct in process and pattern, and we simply have to admit it more openly and take measures in our research to accommodate that state of affairs. Not only do we recognize the multi-level nature of selection in biology, and that an exclusive focus on any higher level of organisation (e.g. cell or organism) will inevitably conceal divergent underlying processes at the genetic level, but we also have begun to acknowledge the diversity of evolutionary processes in action (between eukaryotes and prokaryotes, and within prokaryotes). For prokaryotes, there is an increasing agreement that whenever LGT is frequent enough, trees of genes, genomes, cells, organisms, and perhaps of higher level entities as well, will inevitably diverge. Consequently, as further evidence accumulates, evolutionary biologists will, of necessity, increasingly divorce themselves from traditional tree-monism, even though the monistic principle of descent with modification persists. In practice, we are already studying a diversity of evolutionary processes and considering these as natural, regardless of whether or not our classificatory system consists of only one kind of evolutionary unit (clades). Typically, phylogeneticists are now dealing with a plurality of units in microbial evolution. We need to realize that many of our present "phylogenies of life" correspond to diverse mappings that sometimes represent the history of genes, groups of genes, or perhaps even other categories of entity (for example, processes such as change in genomic G+C content). These different histories do not have to map exclusively or entirely on to one another, but can be acknowledged as evidence of the complexity and richness of microbial evolutionary processes. In that sense, many current tree-rescue efforts are fully consistent with a pluralistic diagnosis. What is not consistent though is the claim that such a tree pattern, when it is found, is a species tree [124], and that it corresponds to the whole of microbial evolution.

All the above has important implications for the "species" notion as well. Rather than working under a single unified concept, microbiologists already accept many different pragmatic definitions of prokaryotic species. They have no species concept that would be relevant for all of life (eukaryotes, let alone prokaryotes) that would justify the reconstruction of a universal species tree. Doolittle and Zhaxybayeva (2009) showed that due to various genetic, population ecological, and evolutionary processes, not all prokaryotes belong to genomically and phenotypically cohesive clusters that biologists could be defined as "species" [125]. In some instances, life-defining processes work together and generate groups of related organisms, sufficiently like one another to be called species. However, the evolution of such coherent clusters is not the general outcome in the prokaryotic world. Rather, various prokaryotic species taxa are defined in nature (and throughout the literature) based on many different criteria, such as global genetic distance (Average Nucleotide Identity, DNA-DNA hybridization experiments) and the presence of some cohesion mechanism (e.g., recombination rates assessed by Multi Locus Sequence approaches, the exploitation of some ecological niche characterized by ecotypes, some phylogenetic inertia). Based on such criteria it is the case that there are multiple correct ways to classify the organic world, and a single organism may be classified in more than one manner depending on the aims of classification.

For instance, two species concepts proposed for prokaryotes are a recombination concept fashioned after the Biological Species Concept [126,127] and the ecotype concept suggested by Cohan [128]. A recent study of the genus *Thermotoga *shows that the same group of organisms forms a single species according to the recombination approach but consists of multiple species according to the ecological approach [129]. Thus each organism in this group belongs to two different types of species (a recombination species and an ecotype species) and those species are not coextensive (having the same spatial and temporal location). In this example, nature imposes a plurality of species concept upon us. The occurrence of lateral gene transfer is also a source of taxonomic pluralism. The recombination concept provides an example. For some microbes, different parts of a single prokaryote genome recombine with different genomes. That is, there is no whole genome recombination in these organisms. The consequence is that by the standards of the recombination concept, the same genome belongs to different species [129]. Similar considerations apply to a phylogenetic approach to classifying microbes. Because of lateral gene transfer (and, as we have noted, due to endosymbiosis in eukaryotes), different parts of an organism's genome often have different evolutionary histories [40,130]. Phylogenetically based classifications for the same group of genomes vary, depending on which clusters of genes in those genomes are chosen. For instance, ribosomal components group the Thermotogales within the bacterial domain as a "basal" branching lineage. If only an unrooted bacterial phylogeny is considered, as seems reasonable because possible outgroups are on very long branches, the Thermotogales appear as a sister group to the Aquificales. In whole-genome phylogenies, the Thermotogales are frequently found to group with Clostridia and Bacilli [131]. Nelson et al. [132] detected many archaeal genes in the genome of *Thermotoga maritima*, a finding supported by the recent analysis of several genomes from members of the Thermotogales [133]. This analysis of five Thermotogales genomes finds that the ribosomal components group Thermotogales with Aquificae. About 8% of the genes group with homologs from Archaea, but the vast majority of genes group with Clostridia homologs. Hence a prokaryote or a part of a prokaryote can belong to more than one classificatory unit and those units do not form a nested hierarchy of inclusive units.

An implication of this discussion is that prokaryotes probably belong to overlapping rather than inclusive hierarchies. In theory, this plurality of definitions of microbial taxa could open the way to multiple classification schemes (i.e., taxonomic pluralism) instead of a single universal hierarchy, often seen as the holy grail of traditional phylogenetics. What are evolutionary microbiologists to make of such pluralism? Should they reject it out of hand given the Linnaean ideal that an organism belongs to only one species and has only one placement in an inclusive hierarchy? Interestingly, the debate over whether to adopt pluralism has already been played out in the general debate of how to define 'species' given the plethora of eukaryote species concepts [134,135]. It shows that adopting a pluralistic approach to microbial taxonomy is not as radical as one might think.

One concern critics of pluralism have is that pluralism lacks a means for distinguishing legitimate from illegitimate classifications [136,137]. They worry that pluralism is too liberal an approach to science because it accepts any suggested classification. That is not the approach being advocated here. Taxonomists stipulate that to be allowed as legitimate, a classification must meet standard scientific criteria [134,138]. And at least one philosopher of taxonomy stipulates that microbial species must be the result of a common type of causal process or be causally efficacious in a similar way [134,138]. For example, if we classify microbes by ecotypes, we need to empirically test whether evolutionary processes cause groups of stable and genetically coherent ecotypes. The same goes for a recombination approach to microbial taxa. If both approaches are empirically confirmed and they cross-cut the world of microbes, then we should allow a plurality of classifications. If one approach is empirically successful and the other fails, then only one of those approaches to microbial classification should be accepted. Taxonomic pluralism is not an *a priori *conjecture but a hypothesis vulnerable to empirical tests.

Another concern with pluralism is whether it leads to inconsistent classifications. As Hennig (1966, 165) writes, "if systematics is to be a science it must bow to the self-evident requirement that objects to which the same label is given must be comparable in some way." [139] If some microbes are grouped according to a recombination species concept and others according to an ecological species concept, then those species are not comparable units. The answer to this concern should not be surprising. Classifications need to be internally consistent, but classifications of different types of entities need not be consistent with one another. Recombination species and ecotype species are different types of entities, bounded by different causal processes, so we should not expect them to be comparable. However, within a particular taxonomic study, if we say there are four species within a genus and three species in another genus, then we had better be comparing like to like. An analogy may help clarify this point. Genera in different phyla (for example, bacterial genera and mammalian genera) are considered very different types of entities. But within a particular classification, genera should be constructed according to the same parameters and thus be comparable.

This still leaves Hennig's concern that a single label is applied to different types of entities. The worry is that the ambiguity of 'species' implied by pluralism leads to semantic confusion [137]. If classifications are constructed according to different parameters and that information is not evident, then we will not know what sorts of entities and relations are represented by a classification. There are two ways to address this concern. One is to get rid of ambiguous terms and replace them with more accurate terms for the different types of units classified. Following the debate over eukaryote species concepts, we might call recombination species 'biospecies,' ecotype species 'ecospecies' and phylogenetic species 'phylospecies.' But the replacement of 'species' with new terms will only go so far once the differences between prokaryote and eukaryote evolution are considered. There are different kinds of ecospecies and biospecies (for example, eukaryotic biospecies whose genomes are involved in whole genome recombination versus prokaryotic biospecies whose genomes recombine in a piecemeal fashion). A more practical approach to avoiding semantic confusion is not to reform our language but to be clear about what type of units are being categorized in a particular classification. For a classification of species, we should say which species approach is being used and how it is being applied (for example, whole genome recombination, or partial genome recombination and which part of the genome). Doing so will avoid semantic confusion and ensure that comparable units are classified within a particular classification.

Stepping back from these details we see that whether or not one should adopt taxonomic pluralism at the species level is largely an empirical question. If nature is cross-cut by significant evolutionary processes, then we should recognize the different types of resultant evolutionary units, whether they are called 'species' or something else. So if we want to accurately describe the species of the microbial world and learn about the processes of microbial evolution, it might be compelling to adopt taxonomic pluralism rather than to stick by default to a single hierarchy.

## Conclusion

There is a longstanding and increasing realization among microbiologists that the mechanisms of gene spread among prokaryotes across evolutionary time are multiple and are different from those of eukaryotes. As a consequence, the gene histories for a large majority of their genes are discordant, which means that the traditional tree of life model is very much a problematic framework to study microbial evolution. Many of the primary tenets and major assumptions of this theoretical framework have been refuted or have undergone drastic modification since its first formulations in Darwin's notebooks. Yet today belief in a single universal tree of life remains largely unaffected, and the strong evidence-driven alternative is often still seen as competition rather than the successor. This persistence of the tree of life model could partly be explained by the fact that it is difficult to fully dislodge an old problematic model without replacing it with a better guiding metaphor. Our discussion above has proposed or implied several potential successors of the tree of life model.

i) A "Central Trend of Life", in which gene transfer also creates the signal. However, any such central trend has to be acknowledged as representing a phenetic and not a cladistic analysis. Although the creation of similarity by gene transfer is a natural process, the reason for phenetic similarity is that successful gene transfer between unrelated organisms is rare [130], and is not due to shared ancestry. This would be unsatisfactory for many evolutionary biologists, eager to learn about the extent of the diversity of processes in microbial evolution.

ii) A "banyan tree" of highly conserved genes, which defines a central trend that is further complicated by extensive LGT. This model appeals to those for whom the large-scale tree-like structure of such a net of life still reflects evolutionary history. From this point of view, phylogenetic reconstruction, especially of reticulation events that connect divergent organisms, is often considered appealing.

iii) A more complex network-like graph in which phases of tree-like evolution (with some horizontal connections) are interspersed with significant phases of rampant horizontal exchange of genetic information. Such processes and their outcomes cannot in principle be represented as trees [140]. Such networks would have the presumed advantage of more fully uncovering the dynamics of prokaryotic chromosome evolution and of providing new insights into the contribution of LGT to microbial evolution. This is probably the position taken by the majority of the authors on this paper.

iv) Radical multiplicity. Some phylogeneticists may prefer not to replace the tree of life by any other unique or dominant "big picture". Such pattern pluralists favour the reconstruction of as many trees and networks as needed to describe the evolution and the structuration of the whole genetic biodiversity. This solution, which leads to a phylogenetic 'forest', seems appropriate to investigate the different evolutionary mechanisms affecting different taxa, at different scales, or for different purposes.

It is clear from some of the analyses discussed above that at least the first two of these four approaches already appeal to the broader community dealing with microbial evolution, even though they continue to use the traditional tree metaphor. Such metaphorical allegiance is likely to continue for quite some time. But given what we now know about prokaryote genome evolution and the contribution of endosymbiosis to eukaryote evolution, it seems rather unlikely that biologists in 20 years will still be using the language of strictly bifurcating trees to describe the relatedness of prokaryotes, and to develop models of microbial evolution.

## Abbreviations

LGT: lateral gene transfer; HGT: horizontal gene transfer; ML: maximum likelihood; NJ: Neighbor-Joining; aa: amino acids.

## Competing interests

The authors declare that they have no competing interests.

## Authors' contributions

All authors substantially contributed to the redaction of the manuscript and have given final approval on the version to be published.

## Reviewers' comments

### Reviewer 1: Ford Doolittle (Dalhousie University)

I have failed in my attempt to identify anything in this paper with which I radically disagree. It presents a temperate account of the current state of the Tree of Life (TOL), for prokaryotes. Indeed, I can only work up some degree of critical fervor by imagining myself in the other camp, for whom a universal tree still deserves a privileged status among possible representations of Life's history. In that assumed role, I'd argue like this.

While endorsing pluralism, authors spend most of their effort in deconstructing tree-monism, rather than allowing it a legitimate if not preeminent explanatory role. In fact a true pluralism must admit that one particular tree-like pattern, which many call the Tree of Cells (TOC, or TOCD&S - Tree of Cell Divisions and Speciations) remains coherent *as a concept*. This TOCD&S would be the tree-like tracing of all cell division events (mostly bifurcations through binary fission but also more complex multifurcative processes by which one cell reproduces its physical self) that have surviving descendants. It would also include speciation events in sexual organisms, which do not reproduce their physical selves but only reproduce their "own kinds". Cells *do *divide, populations *do *split, and lineages of populations that some might want to call species *do *diverge. That polymorphisms persist in recently divided populations (lineage sorting) and that (for bacteria and archaea) recombination falls off at different rates for different genes (and may never reach zero) does mean that branch points in such a TOC are not sharp (Retchless and Lawrence [2007], Science 317: 1093). And of course half of the genes in many bacterial genomes comprise limited distribution "auxiliary" genes that come and go faster than "species" can arise and go extinct. Nevertheless, it still seems sensible to say that *E. coli *K12 and O157:H7 are more closely related to each other than either is to *Yersinia pestis*, and that a rooted tree showing this represents *some *sort of useful historical truth about diverging populations, just as a tree-like pattern that has humans and chimps as a clade to the exclusion of lemurs depicts an important evolutionary reality.

The problems with the TOCD&S are of course that the deeper into the prokaryotic base of it one goes the less it can tell us about the gene content of ancestral genomes (and thus the phenotypes of ancestral cells), the less certainly it can be inferred by *any *averaging or core-gene approach, and the less accurately it represents any kind of genomic history. It may be ultimately unknowable. Still, the inferred TOCD&S is one of many ways to represent data and might be the favored default to serve as reference against which LGTs are displayed. And trying to figure out how to construct it keeps many people employed, generating imaginative new algorithms.

There are two kinds of pluralism to be considered, I think, and the TOCD&S has a role in both. The first kind admits that different models will have different and possibly exclusive applicability in different parts of the biological world (especially, prokaryotes *versus *eukaryotes). The TOCD&S *would *be the TOL for vertebrates (fuzziness at the nodes due to lineage sorting and hybridization aside), but network models would be preferred in representing prokaryotic evolution. The second kind of pluralism allows that we might apply different unitary models to all of biology, recognizing that none is perfect or uniformly relevant across biology, but that each might have its own special value. I still have a hard time ridding myself of the notion that, among this second kind, inclusively hierarchical *classifications *remain especially user-friendly ways of organizing diversity. I don't expect to find copies of the same book on different shelves in my library, or pictures of the same bird on different pages of my bird guide. It's hard for me to see a network as a useful catalog, and so I have no objection to the continued use of an rRNA tree (or of any other agreed upon averaging or gene core-based TOCD&S) as a conventional framework for *classification*, provided everyone knows that that is all that it might be, a conventional taxonomic framework, not the TOL with all its baggage. Other ways of classifying microbes (for instance by gene content or ecological role or indeed by relative position in a multidimensional network) might well have more predictive value, but still this relatively stable hierarchical scheme would serve a very useful organizing function. In fact, I think this is the posture that many microbiologists have already accepted.

I might also accuse the authors of their own unrealistic "ism", prokaryote-eukaryote dichotomization. Like them, I endorse the prokaryote/eukaryote dichotomy as a useful if non-phylogenetic view of the living world, as far as cell structure and the physical processes underlying gene exchange go. But still, there is considerable overlap in what the authors call "mechanisms of natural variation". Although sexual eukaryotes have to recombine to reproduce as organisms, not all eukaryotes are sexual. Some highly recombinogenic bacteria can be treated as effectively sexual in population genetic models, even if they reproduce clonally as organisms. Authors also make too much of eukaryotic recombination being reciprocal: only one product of recombination at meiosis is likely ever to make it into a reproductively successful gamete. And although individual bacteria indeed "non-reciprocally" integrate only a fraction of genomes' information in any single event of legitimate or illegitimate recombination, in the end their population genetics could mimic the eukaryotic situation. It seems to me that we need to exercise explanatory pluralism both within as well as between domains, and that to not do so is a kind of monistic dualism.

I also have a concern over how the authors deal with the perennial skeptic's question, "just how much LGT is there, really". On p. 8, authors say that "it is currently not possible to prove that LGT has not affected all genes in prokaryotes over the full span of evolutionary history that the tree of life purports to depict". Even working my way through the double negative I have some trouble with the concept of "affecting all genes". To me this means that no individual gene in any contemporary genome can be said to have gotten where it is through an unbroken series of genome replications (vertical descent) since the time of whatever is taken as the ancient last universal common ancestor. But to others it might mean that no contemporary *gene family *has fewer than one lateral event in its *entire phylogenetic tree*, even though the majority of lineages one might trace from that ancient time to now are purely vertical. There is a big difference, and yet one sees such ambivalent statements all the time. The authors should commit themselves.

#### Answer to Ford Doolittle

*We thank Ford Doolittle for his very insightful comments. It is certainly helpful to acknowledge that an rRNA tree (or any supposedly representative tree) is a only conventional framework for classification. Although there is no doubt that a tree-like pattern such as the TOCD&S would also be useful, it is questionable whether such a concept is indeed 'coherent', if we have multiple concepts of species, and if cell divisions have to be aggregated into particular groups of cell divisions to make phylogenetic sense rather than anarchy*.

*Certainly there is a lot to be learned about the biology of protists and their evolution. We do not want to ignore them, but we do think that the dualism identified by Doolittle in this paper is currently justified. This heuristic categorization allows us to clarify the deepest issues in the tree of life, since prokaryotes have a significantly non tree-like evolution and, except for endosymbiotic transfers, protists can be conceived to have only a somewhat less tree-like evolution than multicellular eukaryotes. Although we agree with Doolittle that there is certainly some overlap in the mechanisms of genetic variation between eukaryotes and prokaryotes, the biological differences still appear to be of central evolutionary importance to us. Decades of studies have tought us that meiotic recombination is the biologically most important source of genetic variation in eukaryotes. Protists from the same species therefore share the same collection of genes, while prokaryotes may often not. We recognize, however, that not all prokaryotic lineages are similarly affected by lateral gene transfer and recombination, and thus that we should not be rigid in our conceptualization of this duality*.

*Last but not least, we suppressed the double negatives that we incompletely failed not to remove prior to not directly saying what we meant*.

### Reviewer 2: Nicolas Galtier (CNRS, France)

One year ago, Vincent Daubin and I took advantage of an invited article to express our view about the consequences of lateral gene transfers (LGT) and other conflict-generating processes in modern phylogenomics, debating Bapteste's and Martin's (among others) rejection of the tree of life as a useful concept. Then we asked Eric Bapteste for his comments, and he said he would write a full article with appropriate co-authors, which I am now reviewing. So this is my review of a response to our comments on previous articles by these authors, who have the right to reply. Hope we're not boring everybody.

The manuscript is finally not a point-by-point response to the Galtier & Daubin paper, but rather a more general discussion of microbial evolution and systematics. The authors mainly criticize the meaning and usage of a tree of life, as they have done several times in the past, and advocate for 'pluralism", i.e., usage of the appropriate representation/model of the evolutionary pattern/process in specific taxonomic groups, especially prokaryotes (in which LGT is common) vs. eukaryotes (in which it is not).

My feeling about this paper is terribly ambiguous, balancing between almost complete agreement (with the content), and quasi-total rejection (with the form). I shall first comment on what I did not like.

#### Answer to Nicolas Galtier

*We thank Nicolas Galtier very much for his thoughtful comments on our essay. To be clear, what Martin and others reject is the notion that the rRNA tree and 1% representations are graphs from which we can infer the total history of life. For us, the history of prokaryotes is not tree-like in nature, and increasing bootstrap support for branches in trees does not change that circumstance*.

#### Unnecessary contrasts

The discovery of LGT has deeply modified our apprehension of microbial evolution. We now know that a single tree can not be in microbes the unambiguous, meaningful representation of evolutionary history it is in large organisms. LGT weakens the tree of life. Now the question is (I think): does it definitively dismiss it? Should we completely forget about species trees in microbes, or is there still room for this concept, albeit with a modified interpretation? The manuscript implicitly qualifies those who ask this question as "monistic", old-fashionned, dogmatic scientists, whereas those displaying "species tree = devil" on their tee-shirts would be the open-minded, progressist, modern "pluralists". This I think is a caricatural description of the debate and the community. We are collectively facing a practical challenge: how to describe/represent/study microbial evolution and systematics knowing there are frequent LGTs. No need to create spurious "schools of thought", as if a deep philosophical gap was separating two categories of researchers. Please note that creationists are exactly playing this game, calling us the dogmatic, and themselves the pluralists.

#### Answer to Nicolas Galtier

*Our goal was certainly not to polarize the debate with seemingly 'pejorative' terms. We had hoped to provide a useful and thought-provoking description of pluralism and monism, rather than carry out a name-calling exercise. We do not imply that there is a dogmatic divide that cannot be crossed, and we see some very interesting instances in Galtier's response that align nicely with our ideas*.

#### Quantitative vs qualitative

One novelty brought by this manuscript is the notion that because prokaryotes and eukaryotes have distinct cellular machineries with respect to genetic exchanges between cells, their evolutionary histories deserve distinct representations. This probably makes sense. I note, however, that we knew about transformation, conjugation, plasmids and transduction long before the discovery of frequent LGT. At that time, people did not conclude that the evolutionary theory and practice needed to be revolutionized. Only when we discovered discordant gene trees did we start to worry. And if indeed the forthcoming genomes of protist, or fungi, or whatever, reveal strong phylogenetic conflict between genes, we will have to deal with that, whatever the underlying mechanisms. So the "big divide" is perhaps not so relevant, and the problem not so different in distinct groups. The issue is, I think, mostly empirical (what do we do in case of phylogenetic conflict between genes?) and quantitative (up to which level of conflict should we keep drawing "species" trees?).

#### Answer to Nicolas Galtier

*Galtier suggests that almost nobody in the Modern Synthesis worried about prokaryote oddities, but that would seem to have occurred because of the historically deep divide between evolutionary biology and microbiology at that time. Thus, the emphasis should be on discerning who the "we" is that Galtier mentions. LGT was no surprise or problem for microbiologists:, they were familiar with it long before genomics arrived on the biological scene. The discordant genes trees caused phylogeneticists, not microbiologists, to worry*.

*We all agree that such further discordances (if occurring in protists, or fungi) would have to be accommodated, but as noted in the text already, the amount and frequency of such transfer is not yet enough to severely damage the tree structure*.

#### Logical issues

Drawing a prokaryotic tree does not mean rejecting the existence of LGTs, or neglecting their importance, as repeatedly suggested in the text. It means trying to represent the vertical component of the underlying evolutionary process, i.e. inheritance from parent to offspring. This is nonsense only if the vertical component has been entirely erased by massive horizontal tranfers (which might be true in some cases), something the ms fails to demonstrate. The manuscript mokes those who still wish to recover a species tree when genes disagree, for a reason I do not really understand. The metaphor of the American business traveller is a good illustration (p17):

"Averaging the tree signal would be akin to asking about the 'geographic average' destination of an American business traveller, which would probably be (i) somewhere in Iowa, and (ii) would not convey much meaningful information."

First, this comparison is somewhat misleading in suggesting that gene trees are uniformly distributed in the tree space (like the uniformly distributed traveller's location in the USA), which is not true: two gene trees of a prokaryotic data set resemble each other much more closely than two random trees. And genome ("average") trees resemble rRNA trees. More importantly, such trees do not say everything, but they do not say nothing, just like the Iowa location says the traveller works in the USA, which can be good to know in a world-wide context. Of course, the geographic average does not say that the traveller is always located in Iowa, nor does the species tree say that every single nucleotide has evolved according to this topology, as everybody is aware of. The question is "how much does it say?". The tone of the manuscript sometimes suggests the authors think such trees are of no value at all. Conceptually, do they think that vertical inheritance is not a relevant evolutionary process, partly accounting for current microbial diversity, that deserves to be studied and represented? Practically, do they suggest that microbiology would be in better shape if we had, following their recommendation, refused to make use of rRNA trees, mistakenly taken as species trees, during the last 30 years?

#### Answer to Nicolas Galtier

*We repeatedly argued above, and say again now, that a tree capturing the vertical component of evolution is of value, but that it is of limited value because a tree simply cannot show the entirety of prokaryote evolution. That is why reconstructing such a tree must only be attempted critically*.

*The example of the business traveler highlights one of the serious problems of the averaging approach. Given an average location, we cannot distinguish several very different alternatives: that business travelers fly back and forth continually between L.A. and New York, that they visit every state with a particular frequency distribution, or that they spend their entire life in Iowa. The fact that two mutually exclusive alternatives - the traveler has never left Iowa, and the traveler has never been to Iowa - cannot be distinguished, weakens the utility of the averaging approach. Similarly, two divergent phylogenetic 'pulls' can yield an average tree that reflects neither input signal and may thereby present a very misleading view of evolution*.

Furthermore, it is somewhat of an oversimplification to say that genome trees resemble rDNA trees. The extent to which they resemble each other depends strongly on the inclusion or exclusion of closely related lineages. As you travel further back in evolutionary time, all sorts of uncertainties and discrepancies arise: are beta-proteobacteria monophyletic? What about the insect endosymbionts? How about groups like the Spirochaetes, or

*Planctobacteria + Chlamydiales, or the Gram-positives in toto? Is Aquifex an early branch, paired or not paired with Thermotoga, or with the epsilons? All of these variations have been seen in genome trees, and to some extent in 16S trees, depending on rate corrections and compositional recoding*.

Part of the discrepancy perhaps results from the fact that the ms is discussing the "existence" of the tree of life - if it does not "exist" (sensu Woese), then we should forget about it. In my view, a tree is just a human-made conceptual tool that we might decide to adopt if it *means *something to us, like any other graphical representation, irrespective of its "existence" in the real world. That said, I share the authors' concern that the prokaryote tree should not be interpreted the same way as, say, the primate tree, and that it is not a sufficient representation of prokaryote diversity and evolution.

#### Answer to Nicolas Galtier

*We agree. The problem, as we pointed out several times, is when such a tree is used exclusively, and when it is claimed to represent the real and total evolutionary situation*.

#### Conceptual debate, empirical agreement

I found the concluding section of the manuscript remarkably balanced and to-the-point (excluding the very last paragraph). Knowing there are LGTs, what do we do? The authors give 4 options. I think I currently support option 2, which says that we should try to recover the tree representing vertical inheritance, and use it to annotate gene-specific horizontal transfers. The text says a majority of authors support option 3, in which the microbial diversity is represented by a network. I note that these two options are essentially identical, since one natural way to annotate LGT in a species tree is by adding reticulations - perhaps using a distinct colour. So despite the conceptual disagreements I'm expressing above, I end up with similar conclusions/recommendations about what should be done in practice - the really important matter. My only request would be the right for painting in red in the net of life the bifurcating subgraph which traces back vertical inheritance, if identifiable. I hope this is not blasphemy.

#### Answer to Nicolas Galtier

*This most ambitious research program, if conducted critically, is indeed a highly promising line of inquiry to follow*.

### Reviewer 3: John M. Logsdon, Jr. (Dpt. of Biology, University of Iowa, Iowa City, IA 52242 USA)

The prokaryotic tree of life is dead!

The message rings clear in this extraordinary paper from an ensemble group of biologists and philosophers of science. In some ways, I am convinced--and others should be, too. That, I suspect, is the main objective of this paper: to provide the reader with an overwhelming "disproof" of the standard view that prokaryotic evolutionary history occurred as lineage-splitting events and can be depicted by a single bifurcating tree. By interweaving philosophical, technical and empirical arguments, a solid case can be made for inapplicability of traditional tree-thinking and tree-making to prokaryotes. But I also suspect that the larger (and more laudable) goal is to simply challenge the readers' deep-seated sensibilities that such trees must necessarily be at the heart of how we view evolutionary relationships of all organisms.

Prokaryotes *are *different from eukaryotes. Prokaryotes have the luxury of swapping genes (by lateral gene transfer, LGT) between both close and distant relatives, either one gene at time or in large gene sets. This is the crux of prokaryotic sex: the *ad hoc *exchange of genes *via *LGT. Compare this to eukaryotic sex: the equal exchange of whole genomes *via *meiosis. But eukaryotes only exchange genomes between close relatives (generally recognized as "species") and prokaryotes don't need to follow such a rule. The lack of a clear species concept for prokaryotes is a direct consequence of this basic distinction from eukaryotes and is directly implicated in the difficulty (if not inability) to use trees to describe prokaryotic evolution.

Phylogenetic inference has been based on a eukaryo-centric view of evolutionary units (species) that prokaryotes clearly don't follow. In this sense, the authors rightfully argue against a monistic view for understanding evolutionary processes and their resulting histories and favor a pluralistic view that would not be constrained by species and tree thinking, narrowly defined by eukaryotes. It's hard to disagree with this. But whether such processes *completely *undermine the possibility of a prokaryotic tree of life is unclear and worthy of continued analysis and discussion. Although they make a strong case against such a prokaryotic tree, I don't think that these authors will have the last word on this.

Even if they are right about prokaryotes, they seem to paint outside of the lines, in my view, by adding eukaryotic microbes (protists) to their argument: "we should approach the study of microbial (prokaryotes and even possibly protists) genome evolution openly and no longer subordinate our approaches to the study of microbial evolution to the preconceived notion of the tree." This takes the argument too far--there is currently no evidence that eukaryotic microbes experience the same rates and patterns of LGT that would make the standard species concept and resulting tree outcomes like those of prokaryotic microbes. While this is a subtle point; it is important. The frequent reference throughout the paper to "microbes" is misleading and should be changed to "prokaryotes". This paper is about the differences of prokaryotes and eukaryotes and the impacts of these distinctions for (constructing) their evolutionary histories. I argue that this distinction lies mainly in the way in which each lineage has sex. If so, there is reason to think that any major group of eukaryotic microbes is more prokaryotic-like in its sexual predilections than it is standardly meiotic.

In sum, this thought-provoking paper may help to pave a clearer intellectual path for stubborn tree-monists like myself. Although the suggestion of possible successors to the traditional tree of life view (in which I would assign myself to the first or second) is a positive step forward, I have a nagging feeling that in embracing pluralism we just might be missing the actual trees for the forest.

Long live the prokaryotic tree of life!

#### Answer to John Logsdon

*We thank John Logsdon for this very elegant response. It is worth noting that Logsdon, a meiosis expert, agrees with the prokaryote-and-eukaryotes-are-different argument. We agree with him that there is 'currently no evidence that eukaryotic microbes experience the same rates and patterns of LGT that would make the standard species concept and resulting tree outcomes like those of prokaryotic microbes'. Upon his advice, we thus carefully replaced 'microbe/microbial' with 'prokaryote/prokaryotic' where we really did mean only prokaryotes*.

*As for the existence, meaning and usefulness of the prokaryotic tree of life, we also agree we won't have the last word on this debate (but we were not really expecting to). Still, by making the statements above, we feel we have clarified some issues and cleared the ground for addressing what we feel are questions of major importance for evolutionary biology and phylogeny. We are encouraged by all three responses above to think we may have shown how there is room for a diversity of thinking that reflects the diversity of evolutionary processes*.
